# Targeting the MTAP-PRMT5 axis: from synthetic lethality to emerging therapeutic opportunities

**DOI:** 10.1093/oncolo/oyag425

**Published:** 2026-10-22

**Authors:** Vassilis Genoud, Maxime Borgeaud, Mihaela Aldea, Thibaud Koessler, Alfredo Addeo

**Affiliations:** Oncology Department, Geneva University Hospitals, Geneva 1205, Switzerland; Oncology Department, Geneva University Hospitals, Geneva 1205, Switzerland; Department of Medical Oncology, Gustave Roussy, Villejuif 94800, France; Department of Medical Oncology, Gustave Roussy, Villejuif 94800, France; Oncology Department, Geneva University Hospitals, Geneva 1205, Switzerland; Oncology Department, Geneva University Hospitals, Geneva 1205, Switzerland

**Keywords:** MTAP deletion, PRMT5 inhibitors, MAT2A, precision oncology, synthetic lethality

## Abstract

Homozygous deletion of methylthioadenosine phosphorylase (*MTAP)*, one of the most frequent somatic copy-number alterations in cancer, creates a context of synthetic lethal vulnerability. *MTAP* loss leads to the accumulation of 5'-methylthioadenosine (MTA), which partly inhibits protein arginine methyltransferase 5 (PRMT5), and induces a dependency on residual PRMT5 activity; a dependency that can be pharmacologically exploited. This review describes the MTAP-PRMT5 synthetic lethal axis from molecular biology to clinical translation. First-generation nonselective PRMT5 inhibitors were limited by hematological toxicity and reduced potency in MTA-rich environments. Second-generation MTA-cooperative inhibitors, which overcome these constraints by selectively stabilizing the inactive PRMT5-MTA complex, are characterized by tumor selectivity. Early-phase clinical data for MTA cooperative PRMT5 inhibitors showed encouraging signals of efficacy with manageable safety profiles in early-phase trials, establishing clinical proof of concept. Various agents have now entered the next steps of clinical development either alone or in combination. In this review we also discuss the biomarker challenges, essential to patient selection, including the limitations of different methods, as well as emerging resistance mechanisms to PRMT5 inhibitors. Molecular testing standardization, pharmacodynamic endpoint integration, and prospective resistance characterization are identified as priorities for next phases of development of PRMT5 inhibition.

Implications for practice *MTAP* deletion occurs in about 10%-15% of all cancers and offers an opportunity for targeted therapy through PRMT5 inhibition via a mechanism of synthetic lethality. Several MTA-cooperative PRMT5 inhibitors are now advancing in clinical development, alone or in various combinations, and early clinical data seem to support PRMT5 inhibition as a promising approach in *MTAP*-deficient cancers. Beyond confirmation of the clinical benefit of PRMT5 inhibition through larger phase III trials, standardized molecular testing, integration of pharmacodynamic biomarkers, and prospective resistance monitoring will be essential to optimize patient selection and ensure successful clinical implementation.

## Introduction

Loss-of-function alterations are inherently more challenging to treat in oncology because they involve the absence of a gene product, making direct targeting impossible. Yet, such genomic losses are not therapeutically inert, as the molecular and metabolic consequences of a deletion can create collateral dependencies.

The loss of 5′-methylthioadenosine phosphorylase (*MTAP*), one of the most frequent somatic copy-number losses in human cancer, illustrates this principle. MTAP is an essential enzyme in the methionine salvage pathway,^1^ and its loss leads to accumulation of 5′-methylthioadenosine (MTA) in cells, which selectively inhibits the activity of the protein arginine methyltransferase 5 (PRMT5). This creates a dependency on the PRMT5/methionine adenosyltransferase 2A (MAT2A) pathway and a synthetic lethal vulnerability that can be exploited in targeted oncology.

PRMT5 is an arginine methyltransferase with a central regulatory role in cellular biology, modulating epigenetic gene regulation, transcription factor activity, mRNA splicing, and the DNA damage response.^2–10^ Its dysregulation in cancer has been extensively documented, with overexpression acting as a driver of tumor growth and a marker of poor prognosis across multiple tumor types.^11^^,^^12^

Consequently, PRMT5 inhibition has attracted clinical interest. While nonselective PRMT5 suppression is toxic to hematopoietic progenitors, causing hematological toxicities that limited first-generation inhibitors, MTAP deficiency broadens the therapeutic window by rendering tumor cells selectively vulnerable.

This review follows the MTAP-PRMT5 axis from its biological foundations to early clinical trials, molecular testing, emerging resistance mechanisms, and combination strategies.

## The MTAP-PRMT5 axis

### MTAP function

MTAP catalyzes the phosphorolysis of MTA into 5-methylthioribose-1-phosphate, the first step of the methionine salvage pathway that regenerates methionine from polyamine biosynthesis by-products.^13^ As no other mammalian enzyme is known to metabolize MTA, there is a complete absence of functional redundancy.^14^ Consequently, because methionine is an essential amino acid, MTAP-deficient cells become dependent on exogenous methionine supply.^15^

Methionine feeds into the methionine cycle, where it is converted by MAT2A into S-adenosylmethionine (SAM).^16–18^ SAM serves as the universal methyl donor for a vast array of cellular methylation reactions. Among the enzymes that utilize SAM, the PRMT family comprises 9 members, classified into 4 types based on the methylation marks they generate.^19–21^ Type II PRMTs, of which PRMT5 is the principal member, catalyze the symmetric dimethylation of arginine residues (SDMA) on target proteins.^22^ Over 100 PRMT5 substrates have been identified to date, encompassing histones, splicing factors, transcription factors, and proteins involved in DNA damage repair and cell signaling.^23^^,^^24^

A key biochemical aspect driving the MTAP-PRMT5 vulnerability is that MTA and SAM both compete for the same cofactor-binding site on PRMT5.^25^ While SAM enables PRMT5 catalytic activity, MTA binding to the same site inactivates it. Notably, PRMT5 exhibits approximately 40-fold higher affinity for MTA than for SAM.^25^^,^^26^ This difference is unique among methyltransferases and makes PRMT5 highly sensitive to changes in the MTA: SAM ratio inside cells.

SAM also feeds the polyamine biosynthesis pathway through adenosylmethionine decarboxylase 1 (AMD1), generating MTA as a by-product, closing the loop back to the methionine salvage cycle.^14^ The entire pathway is summarized in Figure 1.

**Figure 1.**
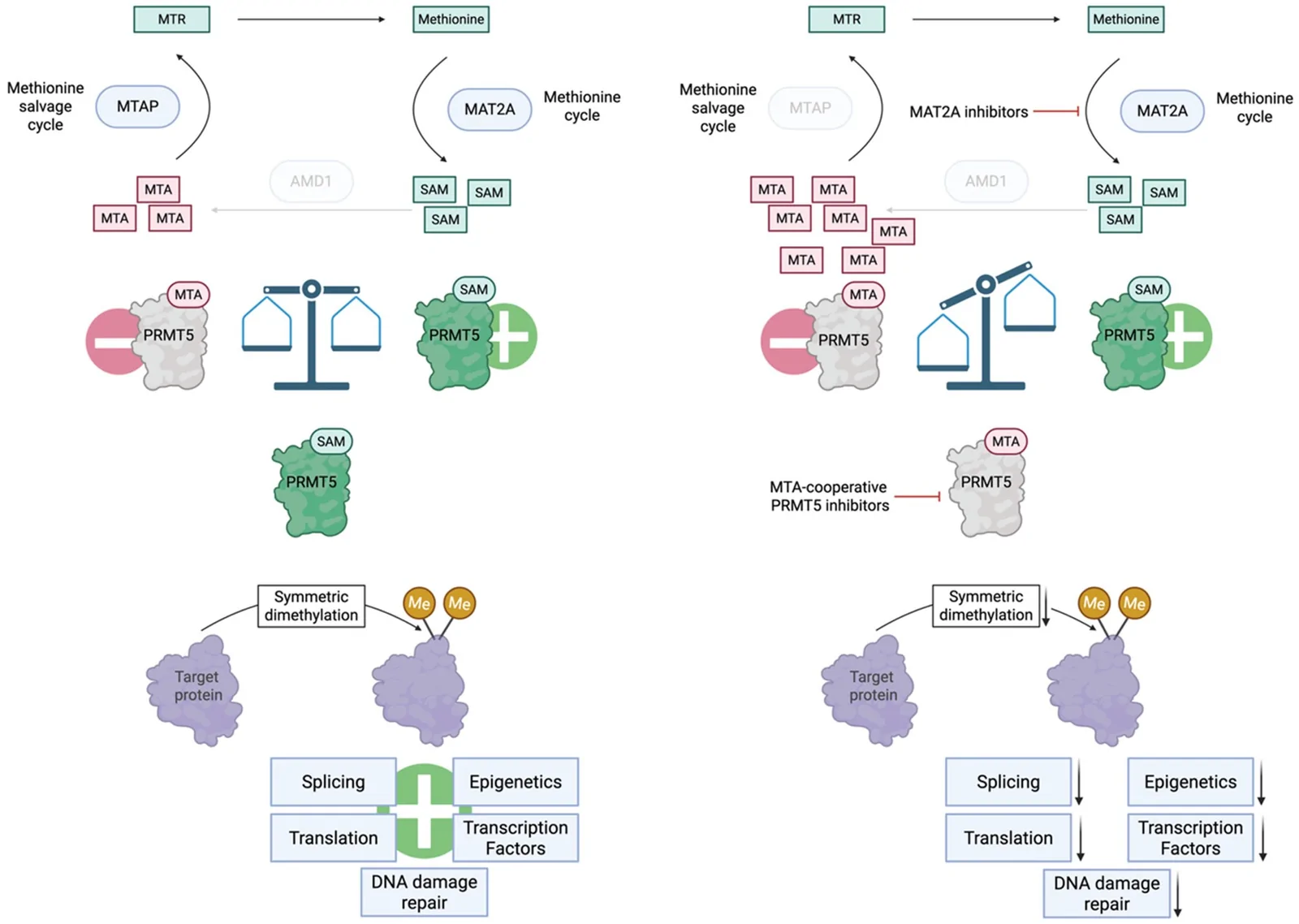
The MTAP-PRMT5 axis under physiological conditions and upon MTAP homozygous deletion. (A) In MTAP-proficient cells, 5′-methylthioadenosine (MTA), a by-product of polyamine biosynthesis via adenosylmethionine decarboxylase 1 (AMD1), is cleaved by MTAP into 5-methylthioribose-1-phosphate (MTR), feeding back into the methionine salvage pathway. Methionine is converted by methionine adenosyltransferase 2A (MAT2A) into S-adenosylmethionine (SAM), the universal methyl donor. SAM binds the cofactor pocket of protein arginine methyltransferase 5 (PRMT5), enabling symmetric dimethylation of arginine residues (SDMA) on histone and non-histone substrates. Under these conditions, intracellular MTA concentrations are low and PRMT5 catalytic activity is maintained (∼74% of baseline), sustaining physiological levels of SDMA on target proteins involved in mRNA splicing, epigenetic regulation, translation, transcription factor activity, and DNA damage repair. Decarboxylation of SAM by AMD1 regenerates MTA, closing the metabolic cycle. (B) Upon homozygous MTAP deletion, MTA cannot be catabolized and accumulates intracellularly. Because PRMT5 has approximately 40-fold lower affinity for SAM compared to MTA, excess MTA displaces SAM from the PRMT5 cofactor pocket, enriching catalytically inactive PRMT5-MTA complexes and reducing global SDMA levels. This partial loss of PRMT5 function renders MTAP-deleted tumor cells dependent on residual PRMT5 activity, establishing the basis for synthetic lethality. Two complementary pharmacological strategies exploit this vulnerability. MTA-cooperative PRMT5 inhibitors selectively bind and stabilize the PRMT5-MTA complex, further suppressing PRMT5 activity specifically in MTAP-deleted cells while sparing MTAP-proficient normal tissues. MAT2A inhibitors deplete SAM production upstream, further increasing the MTA:SAM ratio and favoring the inactive PRMT5-MTA complex. These 2 mechanisms are nonredundant and may be combined to drive PRMT5 activity below the synthetic lethal threshold (≥95% SDMA reduction), resulting in disruption of downstream cellular processes, including RNA splicing, epigenetic regulation, and DNA damage repair. Figure created with BioRender.com.

### Downstream impact of PRMT5

#### Epigenetic regulation

PRMT5-mediated SDMA of histones produces transcriptionally repressive chromatin marks that silence tumor suppressor genes such as PTEN,^3^ and modulate oncogenic programs, including epithelial-mesenchymal transition (EMT).^27^ Beyond cell-intrinsic effects, PRMT5 has been shown to regulate MHC class II expression through histone methylation at the CD74 and CIITA promoters, with potential implications for antigen presentation and immune evasion.^28^

#### Transcription factor regulation

PRMT5 directly methylates several transcription factors with established roles in oncogenesis. Methylation of p53 by PRMT5 attenuates its pro-apoptotic function, as illustrated in hematopoietic stem progenitor cells, where it enhances self-renewal capacity.^29^^,^^30^ PRMT5 also methylates and destabilizes the transcription factor E2F1, promoting cell migration and invasion.^8^^,^^31^^,^^32^ Furthermore, PRMT5 stabilizes c-Myc protein in pancreatic cancer cells, leading to upregulation of the NF-κB pathway.^9^^,^^33^

#### Splicing and DNA damage repair

As an integral component of the spliceosomal machinery,^34^ the loss of PRMT5 promotes aberrant alternative splicing, particularly in pre-mRNAs.^35^ Alternative splicing can activate the p53 pathway, which may serve as a marker of response to PRMT5 inhibitors.^36^ Pharmacological PRMT5 inhibition increases detained introns (DIs), which subsequently reduce protein output.^37^^,^^38^ Such splicing perturbations have been characterized in breast cancer, glioblastoma, and acute myeloid leukemia models.^5^^,^^39^^,^^40^

PRMT5 activity is also tightly coupled to DNA damage repair. Through methylation of specific repair factors, PRMT5 channels double-strand break repair toward the high-fidelity homologous recombination (HR) pathway.^41^ When PRMT5 is inhibited, HR is compromised, and the repair balance shifts toward error-prone nonhomologous end joining.^41^^,^^42^ Pharmacological inhibition of either PRMT5 or MAT2A has been shown to increase hallmarks of unresolved DNA damage.^43^

### Impact of MTAP deletion

The *MTAP* gene is located just 100 kb away from the tumor suppressor gene *CDKN2A*. Due to their genomic proximity, *MTAP* is frequently co-deleted with *CDKN2A*. Early studies reported *MTAP* loss in 80%-90% of cases with *CDKN2A* deletion,^44^ but more recent data suggest a lower rate of approximately two-thirds (66%).^45^ This co-deletion has been consistently associated with adverse prognosis.^46–48^

The prevalence of *MTAP* deletion varies substantially by tumor type. Based on data from the TCGA and other registries, homozygous *MTAP* loss is observed in approximately 10%-15% of all cancers, with enrichment in mesothelioma (∼33%), glioblastoma (∼30%-45%), urothelial carcinoma (∼24%-26%), pancreatic ductal adenocarcinoma (∼18%-22%), cholangiocarcinoma (∼16%), and non-small cell lung cancer (∼14%-15%).^49–54^

Functional studies indicate that *MTAP* itself may act as a tumor suppressor, and its deletion has been linked to disruptions in metabolic, epigenetic, and immune-related signaling pathways, as well as to enhanced cell migration and invasion.^55–60^ 9p21.3 loss has been characterized as a trunk event in cancer evolution, occurring early, prior to additional subclonal diversification.^61^^,^^62^ This clonal architecture implies that *MTAP* deletion is present throughout the tumor, a feature favorable for biomarker-guided therapeutic targeting.

In *MTAP*-deficient tumor cells, the absence of MTA catabolism leads to marked intracellular accumulation of MTA, on average, a 15-fold increase over MTAP-intact cells *in vitro*.^56^ Given the competitive binding of MTA and SAM at the PRMT5 cofactor site and PRMT5′s preferential affinity for MTA,^25^^,^^26^^,^^63^^,^^64^ the excess MTA displaces SAM, enriches inactive PRMT5-MTA complexes, and reduces global SDMA levels.^25^^,^^55^^,^^56^ Importantly, a screen of 33 human methyltransferases demonstrated that the inhibitory effect of MTA is highly selective for PRMT5, sparing other members of the PRMT family (Figure 1B).^25^

This partial loss of PRMT5 activity renders *MTAP*-deleted cells increasingly reliant on the remaining PRMT5 function, forming the conceptual basis for synthetic lethality. PRMT5 is approximately 26% inhibited under physiological conditions, 73% inhibited following *MTAP* loss alone, and up to 93% inhibited when both MTAP and MAT2A are blocked.^26^ Since near-complete PRMT5 suppression (≥95% of SDMA reduction) appears required to trigger synthetic lethality,^65^ this graduated inhibition explains why additional pharmacological intervention is needed to push *MTAP*-deleted cells past the viability threshold.

Additionally, because PRMT5/MAT2A inhibition impairs DNA repair, *MTAP*-deleted tumors may exhibit a functional phenotype resembling homologous recombination deficiency (HRD). This provides a mechanistic rationale for combination with PARP inhibitors, analogous to the synthetic lethal mechanism in BRCA-deficient cancers.^66^

MTAP expression can also be lost without deletion of the locus. In classical Hodgkin lymphoma, MTAP protein is absent from Hodgkin-Reed-Sternberg cells in 84% of primary samples (46/55), and cell lines with low MTAP mRNA show promoter hypermethylation rather than copy-number loss; these models respond to second-generation PRMT5 inhibitors *in vivo*.^67^ What drives the vulnerability is therefore the functional state, MTA accumulation with partial PRMT5 inhibition, rather than the genomic event that produces it. The clinical consequence has now been tested. In the phase I PRIMAVERA study, AZD3470 produced an ORR of 50% among 31 evaluable patients with relapsed/refractory classical Hodgkin lymphoma, rising to 80% at higher doses, with grade ≥3 treatment-related adverse events (TRAEs) in 28% of patients.^68^ Methylation-driven MTAP loss thus extends the eligible population beyond 9p21.3-deleted solid tumors, with the diagnostic caveat discussed below.

## The biomarker challenge

Homozygous *MTAP* deletion is necessary but not sufficient for therapeutic vulnerability. Two challenges complicate the translation of genomic status into patient selection: reliable detection of true homozygous deletion and whether that deletion generates the metabolic phenotype, MTA accumulation, and PRMT5 suppression, on which synthetic lethality depends.

### Homozygous vs heterozygous deletion

Metabolic vulnerability only occurs with homozygous *MTAP* deletion. Heterozygous loss or partial reduction of MTAP still allows enough enzymatic activity to process MTA and keep PRMT5 function above the lethal threshold. Companion diagnostic must therefore distinguish homozygous from heterozygous deletions to identify patients likely to benefit from PRMT5/MAT2A-targeted therapy.^69–71^

### Methods for the detection of MTAP deletion

#### DNA-based methods

NGS with CNV analysis can identify homozygous *MTAP* deletion. As seen above, *MTAP* loss is strongly associated with co-deletion of the neighboring *CDKN2A/B* locus; however, *CDKN2A/B* loss is an imperfect surrogate for *MTAP* deletion as up to 33% of tumors with CDKN2A/B loss can retain an intact *MTAP* gene.^45^^,^^72^ Therefore, direct assessment of *MTAP* status is needed for MTAP-oriented therapies.

However, not every NGS panel is optimal for *MTAP* copy-number assessment. The performance of CNV analysis by NGS depends on panel size and target enrichment strategy, which influence coverage uniformity. Large hybrid-capture-based panels, which enable a more robust copy-number baseline evaluation, allow for a more homogeneous coverage and may be better suited for CNV detection, especially in samples with low tumor purity or genomic complexity.^73^ Accurate CNV calling may be further complicated by low subclonality, aneuploidy, or complex/deeply focal 9p21 events.

Circulating tumor DNA (ctDNA) or liquid biopsy has more limited sensitivity for the detection of CNV, and requires high levels of ctDNA shedding to detect *MTAP* loss. In an analysis with the FoundationOne Liquid CDx assay, *MTAP* loss detection rate with liquid biopsy was comparable to tissue-based assays for tumor fraction >20%.^45^

#### Immunohistochemistry

MTAP immunohistochemistry (IHC) has emerged as a practical complementary approach. Across several tumor types, it has shown good concordance with genomic homozygous 9p21 deletion, though sensitivity and specificity vary by antibody clone, assay conditions, and reference standard.^70^^,^^71^^,^^74^

Protein loss may be easier to recognize than borderline CNV in low-purity specimens, and in NSCLC, some data suggest that IHC may outperform NGS when the panel does not specifically cover *MTAP.*^72^ A key limitation is the absence of harmonized scoring criteria thresholds for the definition of MTAP loss, with cutoff ranging from 0% to <50% of cells retaining MTAP expression across different studies.^72^^,^^75^^,^^76^

Two scenarios produce discordance, both causing sequencing to under-call MTAP deficiency. When *MTAP* is silenced epigenetically, the locus remains intact, so the deficiency is visible by IHC but invisible to copy-number analysis, whatever the panel design.^67^ Additionally, if the panel uses *CDKN2A* loss as a surrogate for *MTAP* loss, it will yield contrasting results, as described in an NSCLC study, in which *CDKN2A* loss identified only 28.4% of IHC-deficient tumors.^72^ IHC and sequencing are therefore complementary rather than interchangeable, as a negative genomic result does not exclude a functionally MTAP-deficient tumor.

## PRMT5 inhibitor mechanisms

### Nonselective PRMT5 inhibitors

The first generation of PRMT5 inhibitors aimed to block enzyme activity regardless of *MTAP* status, as SAM (or substrate) competitive inhibitors.^77–79^ These molecules showed preclinical antiproliferative effects but caused significant toxicity, especially in hematopoietic cells,^80^ leading to grade ≥3 adverse events such as anemia and thrombocytopenia in 23%-51% of patients, with low response rates (0%-7%).^81–86^ All agents impaired T-cell viability, raising concerns about combining with immunotherapy.^87^ A key limitation in MTAP-deleted cells with high MTA is that SAM-competitive inhibitors compete with both SAM and MTA for PRMT5, reducing their effectiveness in target cells.^25^^,^^56^^,^^63^^,^^64^ This prompted the development of a second-generation of MTA-selective agents.

### MTA-cooperative PRMT5 inhibitors

The concept of MTA-cooperative inhibition emerged from the recognition that *MTAP*-deleted tumor cells harbor an excess of PRMT5-MTA complexes not found in normal cells. Rather than competing with MTA, second-generation inhibitors were engineered to bind and stabilize the PRMT5-MTA complex, thereby selectively blocking PRMT5 function only when MTA is present in the cofactor pocket.^65^^,^^88–92^

Key clinical-stage MTA-cooperative PRMT5 inhibitors include BMS-986504 (formerly MRTX1719), AMG193, AZD3470, TNG908 (now replaced by TNG462), and BGB-58067 (Table 1).^65^^,^^88–95^ TNG908 was specifically designed for CNS penetration, given the high prevalence of *MTAP* deletions in glioblastoma, and TNG462 was subsequently optimized for enhanced potency.^90^^,^^94^ Structural studies confirmed that these agents occupy the substrate-binding site when MTA is bound in the cofactor pocket, and all these agents are bioavailable orally.^65^^,^^89^^,^^90^

**Table 1.** Molecules currently in clinical development.

Compound	Mechanism	Trial (NCT)
**BMS-986504**	MTA-cooperative PRMT5 inhibitor	NCT05245500, NCT06883747, NCT06855771, NCT07077434
**AMG193^a^**	MTA-cooperative PRMT5 inhibitor	NCT05094336
**TNG462**	MTA-cooperative PRMT5 inhibitor	NCT06922591, NCT05732831
**TNG456**	MTA-cooperative PRMT5 inhibitor (brain-penetrant, enhanced potency)	NCT06810544
**AZD3470**	MTA-cooperative PRMT5 inhibitor	NCT06130553, NCT06137144
**BGB-58067**	MTA-cooperative PRMT5 inhibitor	NCT07485049, NCT06589596
**IDE892**	MTA-cooperative PRMT5 inhibitor	NCT07277413
**IDE397**	MAT2A inhibitor	NCT07277413
**S095035**	MAT2A inhibitor	NCT06188702
**BG-89894**	MAT2A inhibitor	NCT06568614
**ISM3412**	MAT2A inhibitor	NCT06414460

a AMG193 development has been discontinued (Q1 2026).

In preclinical studies, these compounds demonstrated high selectivity for *MTAP*-deleted over wild-type cell lines (often >70-fold), with dose-dependent SDMA reduction, tumor growth inhibition across xenograft models, and no significant hematologic toxicity.^65^^,^^88–90^^,^^94^ Downstream consequences include disruption of RNA splicing with intron retention, induction of cell-cycle arrest and apoptosis, and impairment of DNA damage repair.^37^^,^^65^^,^^89^

## Early clinical evidence

Phase I data have emerged for AMG193 and BMS-986504 from dose-escalation and expansion cohorts in advanced, pretreated, MTAP-deleted solid tumors, but their interpretation should be approached with caution given the early-phase, nonrandomized, single-arm design.^65^^,^^96^

AMG193 was evaluated in a phase I/Ib trial (NCT05094336) with a maximum tolerated dose of 1200 mg once daily.^65^ Among 74 evaluable patients in the dose-expansion phase, the ORR was 21% at biologically active dose levels, with a DCR of 54.8% and a median duration of response (DOR) of 8.3 months. Responses were observed across NSCLC, pancreatic adenocarcinoma, biliary tract cancer, melanoma, and other tumor types. Pharmacodynamically, near-complete SDMA suppression was observed at clinically active doses. Nevertheless, Amgen announced the discontinuation of AMG193 at its Q1 2026 Earnings Conference Call, without presenting new clinical data, highlighting the challenge of translating early phase I signals into clinically meaningful benefit.

In the phase I dose-escalation study (NCT05245500), BMS-986504 (navlimetostat) was evaluated across doses up to 800 mg once daily, with a maximal acceptable dose of 600 mg once daily.^96^ Among 147 patients, the safety profile was manageable, with ≥3 TRAEs in 12%, and led to discontinuation in only 3% of patients. Importantly, hematologic toxicity, which had previously been a key limitation of nonselective PRMT5 inhibitors, was infrequent, with anemia observed in 10% of patients (2% at grade ≥3). Among 124 evaluable patients, activity was observed in NSCLC (ORR 31%), mesothelioma (ORR 36%), pancreatic cancer (ORR 15%), and cholangiocarcinoma (ORR 18%).^96^ Interestingly, the median time to response was 4.5 months, and 88% of responding patients initially achieved stable disease on the first evaluations, prior to tumor regression, a pattern consistent with the hypothesis that sustained target engagement and progressive epigenetic reprogramming are required for therapeutic effect, although prospective validation of this interpretation is needed. Pharmacodynamic analyses confirmed strong and dose-dependent target engagement, with marked reductions in tumor SDMA levels and increased intron retention.^43^^,^^97^

In contrast, clinical data for other agents remain limited. TNG908 failed to demonstrate meaningful activity in glioblastoma, with no objective responses observed in early analyses, leading to a strategic shift toward TNG462 and TNG456. For TNG462 (vopimetostat), preliminary data reported an ORR of 27% and a median progression**-**free survival of 6.4 months among 94 patients; however, peer-reviewed publication is awaited.

Collectively, these phase I data establish a clinical proof of concept for MTA-cooperative PRMT5 inhibition, with response rates of approximately 20%-35% in the most responsive tumor types and a safety profile that overcomes the hematological toxicity that limited first-generation agents. However, small cohort sizes, absence of randomized controls, and immature survival data need confirmation. Ongoing phase II/III trials, such as the MountainTAP-30 study in pancreatic cancer, are awaited to clarify clinical benefit.

## Resistance

Resistance to MTA-cooperative PRMT5 inhibitors remains poorly characterized, reflecting the novelty of the drug class and limited follow-up in current phase I cohorts.

Primary resistance could be driven by stromal MTA clearance or heterogeneous intratumoral MTA accumulation, thereby attenuating the synthetic lethal threshold in a subset of genomically *MTAP*-deleted tumors.

Secondary or acquired resistance could also occur, such as compensatory upregulation of the MAT2A protein after MAT2A inhibition, partially restoring SAM biosynthesis and reducing PRMT5 suppression. This mechanism appears specific to MAT2A inhibitors and has not been observed with MTA-cooperative PRMT5 inhibitors, which do not directly affect MAT2A or PRMT5 expression.^98^ This distinction has direct implications for combination design, as pairing MAT2A inhibitors with MTA-cooperative PRMT5 inhibitors may mitigate MAT2A upregulation by targeting PRMT5 simultaneously through an independent mechanism.

Secondary mutations in PRMT5 at or near the MTA-binding pocket represent another potential resistance mechanism, analogous to those conferred by allosteric inhibitors in other oncological contexts. Structural alterations that reduce MTA affinity, disrupt inhibitor binding, or decouple MTA occupancy from catalytic inactivation could selectively restore PRMT5 function in tumor cells. This mechanism is theoretical and has not yet been reported.^98^

Metabolic rewiring of the methionine salvage pathway represents a further theoretical route: upregulation of alternative methionine sources or downregulation of AMD1 could reduce the MTA: SAM ratio or limit the MTA pool on which cooperative inhibitors depend, though neither has been characterized in clinical samples.^99–101^

Additionally, tumor cells may escape the biological consequences of PRMT5 inhibition by activating complementary oncogenic survival pathways. Preclinical evidence from *in vitro* resistance models demonstrates that acquired resistance to MTA-cooperative PRMT5 inhibition is not explained by restoration of PRMT5 activity but associates with upregulation of MAPK transcriptional programs and collateral MEK sensitivity,^99^ illustrating how tumor cells could bypass the PRMT5 pathway by activating other oncogenic pathways. Other more theoretical mechanisms, such as secondary mutations affecting the MTA-binding pocket, have not been observed.

To address these potential resistance pathways, incorporating resistance-monitoring strategies into the design of expansion cohorts and randomized trials could substantially improve our knowledge of the MTAP-PRMT5 axis. Serial SDMA levels, ctDNA tracking of clonal evolution, and biopsies at progression will be needed to characterize the resistance landscape as the field matures.

## Combination strategies

### Combination with MAT2A inhibitors

An alternative approach to exploit the MTAP-PRMT5 axis targets the upstream enzyme MAT2A. By inhibiting SAM biosynthesis, MAT2A inhibitors reduce the methyl donor available to PRMT5 while simultaneously allowing MTA to dominate the cofactor pocket.^25^^,^^43^ Because the inhibitory effect on PRMT5 is gated by the MTA: SAM ratio, MAT2A inhibition is inherently more potent in *MTAP*-deleted cells, where MTA is already elevated, providing a natural therapeutic window.^25^

The clinical-stage MAT2A inhibitors AG-270/S095033 and IDE397 reduce plasma SAM levels in a dose-dependent manner regardless of *MTAP* status, but SDMA reduction is observed selectively in MTAP-deleted models.^43^^,^^100^^,^^101^

AG-270/S095033 showed limited clinical efficacy (DCR 17.5%) in a phase I trial, leading to the discontinuation of its development in favor of the next-generation inhibitor S095035.^102^

Given their independent mechanisms of action, combining MTA-cooperative PRMT5 inhibitors with MAT2A inhibitors is an attractive strategy. Preclinical data demonstrate synergistic anti-tumor activity.^103^ The combination of IDE397 and AMG193 has been evaluated in a clinical trial that has completed enrollment (NCT05975073), and S095035 is being evaluated in combination with TNG462 (NCT06188702). Whether the combination adds to dose optimization of either agent alone remains unknown.

### Combinations with chemotherapy

The impairment of DNA repair induced by PRMT5/MAT2A inhibition provides a mechanistic rationale for combining these agents with DNA-damaging agents. Preclinical evidence suggests a synergistic effect with taxanes, platinum-based chemotherapy, and antifolates such as pemetrexed.^43^^,^^104^ Notably, resistance to PRMT5 inhibition correlates with increased expression of stathmin 2 (STMN2), a microtubule regulator, rendering resistant cells more sensitive to taxanes.^43^ The randomized phase II/III MountainTAP-30 (NCT07076121) study is evaluating BMS-986504 vs placebo in combination with nab-paclitaxel and gemcitabine in patients with metastatic, *MTAP*-deleted, pancreatic adenocarcinoma.

### Combinations with immunotherapy

The interplay between PRMT5 and anti-tumor immunity is complex and potentially bidirectional. PRMT5 dampens the interferon-γ response by methylating innate immune sensors and suppressing MHC class I gene transcription.^105^ Pharmacological PRMT5 inhibition reverses these effects, enhancing type I interferon production and upregulating antigen presentation machinery.^105^ In a hepatocellular carcinoma mouse model, PRMT5 inhibition increased lymphocyte infiltration and MHC class II expression; combination with anti-PD-1 therapy achieved significantly greater reductions in tumor volume and greater T cell infiltration than either agent alone.^28^

These immunostimulatory effects are, however, counterbalanced by opposing signals. PRMT5 inhibition has been shown to increase PD-L1 expression in lung cancer models,^106^ which might reduce anti-tumor immunity while simultaneously providing a rationale for anti-PD-L1 combination. The MTA-rich microenvironment of *MTAP*-deleted tumors independently promotes immunosuppression, as MTA activates the adenosine A2B receptor on tumor-associated macrophages, promoting immunosuppressive M2 polarization,^58^ and tumor-secreted MTA/SAM decreases chromatin accessibility in infiltrating T cells, leading to exhaustion.^107–109^ Prospective immunological endpoints in ongoing combination trials are therefore needed.

Several ongoing trials are evaluating PRMT5 inhibitors in combination with immunotherapy. The phase III MountainTAP-29 study (NCT07063745) evaluates BMS-986504 vs placebo, with pembrolizumab and platinum-based chemotherapy for NSCLC with *MTAP* deletion in the first-line setting. The phase Ib MTAPESTRY-104 (NCT06333951) trial was similarly evaluating the combination of AMG193 with pembrolizumab alone or pembrolizumab-chemotherapy in the first-line setting for NSCLC.

### Combinations with targeted therapies

*MTAP* loss may co-occur with oncogenic driver alterations across multiple tumor types. In NSCLC, *MTAP* loss frequently co-occurs with oncogenic drivers, particularly *EGFR* mutations (*EGFR* mutations are found in 48% of MTAP-loss NSCLC).^53^^,^^54^^,^^75^  *MTAP* deletion seems to modestly impair TKI efficacy, PFS, and time under treatment with osimertinib monotherapy, but without affecting overall survival.^53^^,^^75^ Acquired MTAP loss has been reported to emerge in 13% of resistant cases under targeted therapy.^53^ This dual-alteration pattern has prompted interest in combination strategies, targeting both the PRMT5/MAT2A axis and the oncogenic driver.^75^  *In vitro* studies suggest a potential synergy between EGFR/ALK and PRMT5 inhibition in *EGFR*/*ALK* NSCLC models, but this remains exploratory.^75^

In *KRAS*-mutated tumors, the co-occurrence of *MTAP* deletion is of particular interest, especially in pancreatic adenocarcinoma. Preclinical data support synergy between PRMT5 inhibitors and KRAS blockade,^110^ and this combination is now under early clinical evaluation. TNG462 is currently being tested in phase I/II with RAS inhibitors (NCT06922591), with preliminary results from company announcements suggesting a high response rate of 92% (amongst only 12 response-evaluable patients) for the combination of TNG462 and daraxonrasib in *KRAS*-mutated *MTAP*-loss pancreatic cancer, though peer-reviewed data remain pending.^111^ BMS-986504 is being tested concurrently with the RAS (ON) inhibitor RMC-6236 in the MountainTAP-5 (NCT07492680) studies.

Ongoing combination trials are summarized in Table 2.

**Table 2.** Combination trials in clinical development.

Combination	Strategy	Trial (NCT)
**BMS-986504 + pembrolizumab + platinum-based chemotherapy**	PRMT5i + chemo-IO	NCT07063745 (MountainTAP-29)
**BMS-986504 + nab-paclitaxel + gemcitabine**	PRMT5i + chemotherapy	NCT07076121 (MountainTAP-30)
**BMS-986504 + RMC-6236**	PRMT5i + RAS(ON) inhibitor	NCT07492680 (MountainTAP-5)
**AMG193^a^ + docetaxel**	PRMT5i + chemotherapy	NCT05094336 (MTAPESTRY-101)
**AMG193^a^ + pembrolizumab ± chemotherapy**	PRMT5i + chemo-IO	NCT06333951 (MTAPESTRY-104)
**AMG193^a^ + chemotherapy (gem/nab-P or mFOLFIRINOX)**	PRMT5i + chemotherapy	NCT06360354 (MTAPESTRY-103)
**AMG193^a^ + IDE397**	PRMT5i + MAT2Ai	NCT05975073
**TNG462 + RAS inhibitors**	PRMT5i + RAS inhibitor	NCT06922591
**TNG462 ± S095035**	PRMT5i ± MAT2Ai	NCT06188702
**IDE892 + IDE397**	PRMT5i + MAT2Ai	NCT07277413

a AMG193 development has been discontinued (Q1 2026).

## Perspectives

The field is now shifting from proof of concept to clinical consolidation. Multiple related questions will influence whether the therapeutic potential of MTAP-directed synthetic lethality is achieved.

Integration of pharmacodynamic biomarkers into trial design is the most needed methodological improvement. Companion diagnostic standardization, with harmonized IHC MTAP loss thresholds and validated NGS-based CNV criteria, is a prerequisite for phase II trials and potential regulatory approval. Incorporating SDMA levels or MTA into ongoing randomized studies would link target engagement to outcomes and identify patients with a confirmed biological vulnerability rather than genomic eligibility only. Stroma-rich tumor types, such as glioblastoma or pancreatic adenocarcinoma, may require dedicated validation of the metabolic phenotype, given the potential stromal MTA sink effect described above.

Systematic characterization of resistance is also urgently needed. The mechanisms outlined above have not yet been studied in patients progressing on MTA-cooperative PRMT5 inhibitors. Prospective re-biopsy at progression, potentially combined with ctDNA monitoring for clonal evolution, should be embedded in trials as standard practice.

Third, the sequencing and prioritization of combination approaches requires clarification. While preclinical data support multiple combination strategies, clinical development will need to prioritize the most promising for exploration. The slow response kinetics of BMS-986504, with a median time to response of 4.5 months and 88% of responders initially presenting with stable disease,^96^ raises important questions for combination trial design: standard early progression criteria may prematurely exclude responding patients, potentially requiring adapted evaluation schedules.

Finally, tumor-type prioritization will shape the next development phase. Mesothelioma, pancreatic adenocarcinoma, and glioblastoma share high *MTAP* deletion prevalence with limited standard-of-care options, though glioblastoma presents specific challenges of blood-brain barrier penetration and stromal MTA clearance. The intersection of *MTAP* deletion with co-occurring driver alterations, particularly in *KRAS*-mutant pancreatic tumors and oncogene-addicted NSCLC, may define subsets where rational combinations offer the greatest leverage.^75^^,^^110^

## Conclusions

Homozygous *MTAP* deletion, long considered a bystander consequence of 9p21.3 loss, has emerged as a therapeutically actionable vulnerability. The accumulation of MTA in *MTAP*-deficient cells selectively compromises PRMT5 function, creating a metabolic dependency that can be exploited through synthetic lethality.

MTA-cooperative PRMT5 inhibitors have demonstrated clinical proof of concept in early-phase trials, with response rates of approximately 20%-30% in the most responsive tumor types and manageable safety profiles. These results, derived from single-arm, phase I extension cohorts, established a signal that now requires confirmation in randomized, adequately designed, and powered studies. Multiple agents are advancing into randomized trials, including combinations with MAT2A inhibitors, chemotherapy, immune checkpoint inhibitors, and targeted therapies.

The biomarker challenge, confirming that genomic deletion is biologically active, and the mechanisms of therapeutic escape are not peripheral concerns but central determinants of clinical applicability. Standardization of companion diagnostics, integration of pharmacodynamic endpoints in trial design, and systematic characterization of resistance in prospectively collected progression biopsies are the methodological priorities that will define the next phase of clinical development.

*MTAP* deletion occurs in about 10%-15% of all cancers and is more common in tumor types with high unmet therapeutic needs. Whether MTA-cooperative PRMT5 inhibitors ultimately fulfill their promise will depend on the potency of these agents as much as on precise patient selection.

## Data Availability

No new data were generated or analyzed for this review. All data discussed are available in the cited publications.
